# Reduced Apolipoprotein Glycosylation in Patients with the Metabolic Syndrome

**DOI:** 10.1371/journal.pone.0104833

**Published:** 2014-08-12

**Authors:** Olga V. Savinova, Kristi Fillaus, Linhong Jing, William S. Harris, Gregory C. Shearer

**Affiliations:** 1 Cardiovascular Health Research Center, Sanford Research USD, Sioux Falls, South Dakota, United States of America; 2 Department of Chemistry and Biochemistry, South Dakota State University, Brookings, South Dakota, United States of America; 3 Department of Internal Medicine, Sanford School of Medicine, University of South Dakota, Vermillion, South Dakota, United States of America; University of Basque Country, Spain

## Abstract

**Objective:**

The purpose of this study was to compare the apolipoprotein composition of the three major lipoprotein classes in patients with metabolic syndrome to healthy controls.

**Methods:**

Very low density (VLDL), intermediate/low density (IDL/LDL, hereafter LDL), and high density lipoproteins (HDL) fractions were isolated from plasma of 56 metabolic syndrome subjects and from 14 age-sex matched healthy volunteers. The apolipoprotein content of fractions was analyzed by one-dimensional (1D) gel electrophoresis with confirmation by a combination of mass spectrometry and biochemical assays.

**Results:**

Metabolic syndrome patients differed from healthy controls in the following ways: (1) total plasma - apoA1 was lower, whereas apoB, apoC2, apoC3, and apoE were higher; (2) VLDL - apoB, apoC3, and apoE were increased; (3) LDL - apoC3 was increased, (4) HDL -associated constitutive serum amyloid A protein (SAA4) was reduced (p<0.05 vs. controls for all). In patients with metabolic syndrome, the most extensively glycosylated (di-sialylated) isoform of apoC3 was reduced in VLDL, LDL, and HDL fractions by 17%, 30%, and 25%, respectively (p<0.01 vs. controls for all). Similarly, the glycosylated isoform of apoE was reduced in VLDL, LDL, and HDL fractions by 15%, 26%, and 37% (p<0.01 vs. controls for all). Finally, glycosylated isoform of SAA4 in HDL fraction was 42% lower in patients with metabolic syndrome compared with controls (p<0.001).

**Conclusions:**

Patients with metabolic syndrome displayed several changes in plasma apolipoprotein composition consistent with hypertriglyceridemia and low HDL cholesterol levels. Reduced glycosylation of apoC3, apoE and SAA4 are novel findings, the pathophysiological consequences of which remain to be determined.

## Introduction

The prevalence of metabolic syndrome (MetSyn) in the US and worldwide remains high [1]–[5] and is associated with the increased cardiovascular disease (CVD) risk [6], [7]. The diagnosis of MetSyn is based on the presence of any three or more components from the list of central obesity; reduced high density lipoprotein cholesterol (HDL-C); elevated plasma triglycerides (TG) or glucose; or high blood pressure [8].

Apolipoproteins (apo) are structural components of lipoprotein particles that also direct particle metabolism [9], [10]. The levels of apoB (found in very low, low and intermediate density lipoproteins; VLDL, LDL, and IDL, respectively) and apoA1 (in HDL) and their ratio can serve as markers of CVD risk [11], [12]. Moreover, increased exchangeable apoC3 content in LDL is associated with increased CVD risk [13]. A perturbation in apo levels would not be surprising in MetSyn given the fact that apoB is closely associated with abdominal obesity and other features of MetSyn [14]. Indeed, apoB significantly and independently predicted CVD risk among post-infarction patients with metabolic syndrome [15]. Plasma levels of apoC3 and apoE were also shown to be elevated in MetSyn [16], [17]. Some evidence of apo composition of lipoprotein classes in MetSyn is available from the proteomic studies: thus, HDL (HDL3 subclass) isolated from dyslipidemic subjects (with new diagnosis of coronary artery disease) were reported to be enriched in apoE [18]; whereas LDL (small dense LDL subclass) from a different cohort of subjects with MetSyn were enriched in apoC3 and depleted of apoC1, apoA1, and apoE compared with matched healthy controls [19].

The functions of apolipoproteins are likely to be affected by posttranslational modifications. In type 2 diabetes, for example, apoB can become damaged by glycation and oxidation [20], but less is known about endogenous glycosylation of apos in disease states. ApoC3 and apoE are modified by mucin-type O-linked glycosylation during transit through the Golgi, where carbohydrate chains are enzymatically attached to specific serine or threonine residues in these proteins [21]–[26]. Sialic acids (amino sugars) are commonly found as terminal oligosaccharide residues on O-glycosylated lipoproteins including apoC3 and apoE [27]. ApoC3 is present in three isoforms: a minor non-glycosylated isoform (commonly denoted apoC3-0), and two glycosylated isoforms, which differ in the number of sialic acid residues attached (mono-sialylated and di-sialylated isoforms, apoC3-1 and apoC3-2). These sialo-isoforms can be resolved by 1D electrophoresis. ApoE also exists in multiple glycol-isoforms (up to eight isoforms were detected by mass spectrometry), which vary in charge because of variable sialylation [22]. 1D electrophoresis typically resolves non-glycosylated apoE (low molecular weight (MW) isoform) and glycosylated apoE (high MW isoform); and 2D electrophoresis is required to resolve sialo-isoforms of apoE [28]. Similarly, serum amyloid A-4 protein (SAA4), a constitutively expressed protein associated with HDL, is partially modified by N-glycosylation resulting in two major isoforms, low MW non-glycosylated isoform (∼14 kD) and high MW glycosylated isoform (∼19 kDa) containing variable numbers of sialic acid residues [29], [30].

Sialylation of lipoproteins appears to be protective against coronary heart disease (CHD) since lipoproteins isolated from healthy individuals contain more apo-associated sialic acid compared to subjects with atherosclerosis [31]–[34]. Reduced glycosylation of apoE is implicated in preeclampsia [35], whereas changes in apoC3 glyco-isoform ratio (mono-sialo/di-sialo) have been observed in uremia [36], kidney disease [37], and several other pathologies including obesity, in which mono-sialo/di-sialo apoC3 ratio positively correlated with body mass index (BMI) before and after bariatric surgery, suggesting that sialylation of apoC3 is reduced in obese individuals [38].

The purpose of this study was to describe in detail the differences in apo composition of the three major lipoprotein families in patients with MetSyn as compared to healthy controls. Differences in apo composition, and possibly in apo glyco-isoform ratios, could expand our understanding of the pathology underlying MetSyn.

## Methods

### Ethics statement

Human study protocols were approved by the Institutional Review Board of the University of South Dakota, and by the Institutional Review Board of the Sanford Health. Written informed consent was obtained from all study participants.

### Human plasma samples

MetSyn subjects (n = 60) were recruited, assessed and treated as previously described [48]. This study was registered at clinicaltrials.gov (NCT00286234). Control subjects (n = 14) were recruited as a comparator group to match the age and the sex distributions in MetSyn group. The protocol was approved by the Institutional Review Board of the University of South Dakota.

MetSyn subjects were enrolled between December 2007 through April 2008. Inclusion criteria for the MetSyn subjects were body mass index (BMI) greater than 25 kg/m^2^, TG greater than 150 mg/dL, and a TG/HDL-C >3.5. In the original study 60 MetSyn subjects were included. In the present report, four subjects were excluded because of an insufficient amount of plasma for lipoprotein preparation or a technical failure in the lipoprotein preparation procedures. Fourteen control subjects were recruited between July and September of 2008 to match the age and the sex distributions in MetSyn group. Inclusion criteria for control group were BMI less than 25 kg/m^2^; TG less than 150 mg/dl; and HDL-C greater than 50 mg/dl.

Plasma was isolated within 1 hour of collection and plasma samples were stored at −80°C before preparative sequential ultracentrifugation. The secondary analysis of de-identified human plasma samples was approved by the Institutional Review Board of the Sanford Health.

### Lipoprotein preparations

Lipoproteins were isolated from EDTA plasma by sequential ultracentrifugation in densities 1.006; 1.063; and 1.21 g/ml corresponding to VLDL, IDL/LDL, and HDL fractions as previously described [39], and stored frozen (−80°C) until analysis.

### 1D electrophoresis

Lipoprotein fractions (4.5 µg of protein) were subjected to gradient SDS-PAGE (4–20% Peptide gels, BioRad, Hercules, CA) and stained with Sypro Orange (Invitrogen, Grand Island, N.Y.). Gels were scanned using Typhoon scanner at 532/555 nm excitation/emission wave lengths and analyzed using Image Quant version 5.0. Our prior studies have shown that this method produces quantitative results comparable with LC-MS methods of quantitation [40]. To further add to the consistency of lipoprotein fraction, we employed balanced batching and quality controls. Intensities of all bands were measured as area under the curve with baseline adjusted manually. The absolute amount of protein in each band was calculated based on its fraction of total protein loaded (4.5 µg per lane). The lowest amount of protein detected in a band was about 5 ng and the highest was about 1000 ng, which are within linear range of detection for Sypro Orange protein stain, 2–2000 ng per band (Fluorescence Imaging: principles and methods, Amersham Biosciences, code number 63-0035-28, (2000)). The concentrations of each apo in total plasma were determined by summing the concentrations in VLDL, LDL, and HDL obtained from sequential ultracentrifugation.

### Apolipoprotein identification

#### 2D electrophoresis

This was performed with a single VLDL and HDL samples to aid with protein identification. Tentative identifications of major bands in VLDL and HDL fractions were assigned by the comparison between their migration on 1D and 2D gels based on published 2D results from others [41], [42].

#### Protein identification by nanoLC-MS/MS

This was performed at Sanford-Burnham Institute for Medical Research (SBIMR, La Jolla, CA) proteomic CORE following standard protocol. Briefly, protein bands are excised from Coomassie-stained gels and digested with Trypsin and purified using C18 ZipTip (Millipore, Inc.). Samples were resolved on a reversed phase column (15 cm in length, 100 mm id) packed with 5 µm diameter Magic C18 AQ resin (Michrom) using a linear gradient elution from buffer A (2% acetonitrile in H2O plus 0.1% formic acid) to 15% buffer A plus 85% buffer B (acetonitrile plus 0.1% formic acid) in 45 min. The Eksigent Nano 2D LC system was equipped with an ADVANCE ESI source (Michrom). LC/MS was operated in the data dependent mode. The MS/MS spectra were analyzed by Sorcerer Enterprise v.3.5 (Sage-N Research Inc.) with SEQUEST algorithm as the search program for peptide/protein identification. SEQUEST was set up to search the target-decoy ipi.HUMAN.v3.73 and ipi.RAT.v3.73 databases containing protein sequences using trypsin for enzyme. The search results were viewed, sorted, filtered, and statically analyzed by proteomics data analysis software, Peptide/Protein prophet v.4.02 (ISB).

#### Peptide mapping of apoB isoforms by nanoLC-MS/MS

This was performed at South Dakota State University (SDSU, Brookings, SD). ApoB and its suspected truncated isoform bands were digested by trypsin. The in-gel digested peptides were brought up in 0.1% formic acid. The peptides were purified on C18 IntegraFrit Sample trap (New Objective, Inc., Woburn, MA) and resolved on a C18 PicoFrit Analytical Column (New Objective, Inc., Woburn, MA) using Eksigent nanoLC system with a multistep gradient of solvent 2A (water premixed with 0.1% formic acid) and solvent 2B (acetonitrile premixed with 0.1% formic acid). The LC-MS/MS raw data was obtained using LTQ- mass spectrometer (Thermo Scientific, San Jose, CA), converted to DTA files using Thermo Scientific Proteome Discoverer, and correlated to theoretical fragmentation patterns of tryptic peptide sequences from the Uniprot-Sprot.fasta database using SEQUEST. Search parameters included (1) variable modifications allowing mass increase of 57 Da for possible carbamidomethyl-modified cysteines and 18 Da for oxidized methionines; (2) restricted to trypsin generated peptides, allowing for two missed cleavages; (3) the criteria for peptide were based on top hit(s) with individual cross correlation exceeding a threshold dependent on the precursor charge state.

#### MALDI-TOF analysis of apoC3 glyco-isoforms

This was performed at South Dakota State University (SDSU, Brookings, SD) mass-spectrometry facility following published protocol [38]. Briefly, lipoprotein samples (0.5 µL) were diluted with water:acetonitrile:trifluoroacetic acid (TFA) (15 µL, 95∶5:0.1), and extracted with a C4 ZipTip (Millipore, Inc.) or C18 mini spin column (Pierce, Inc.). 0.75 µL of the eluted sample in water:acetonitrile:TFA (25∶75:0.1) was applied to the MALDI target along with sinapinic acid (0.75 µL saturated solution in water:acetonitrile:TFA, 50∶50:0.1). Uniform crystallization was achieved by manual mixing of the sample until crystals were formed. Samples were dried and analyzed in a Bruker Daltonics Biflex IV MALDI-TOF mass spectrometer operated in the linear mode. The spectrum for each samples were collected with 500 laser shots. The spot of laser impact was also changed frequently during each data acquisition.

#### Western blotting

This was performed following standard protocols [43]. Briefly, 5µg of LDL and HDL samples were resolved on 5% SDS-PAGE gel and transferred to PVDF membrane. Membrane was blocked in 5% bovine serum albumin (BSA) and incubated with apoB antibody specific to amino-terminal human apoB peptide (Santa Cruz Biotechnologies) followed by peroxidase-conjugated secondary antibody. Specific bans were detected by measuring chemiluminescence using an ECL Advanced Western Blotting Kit (GE Healthcare, Piscataway, NJ).

#### Enzymatic removal of sialic acid residues

VLDL and HDL preparations were incubated with neuraminidase, a glycoside hydrolase enzyme (EC 3.2.1.18) that cleaves the glycosidic linkages of sialic acid (NE Biolabs), at 1 U enzyme:1 mg protein ratio in 50 mM sodium citrate buffer, pH 6.0, for 15 minutes at 37C followed by SDS-PAGE separation.

### Statistics

GraphPad Prism version 5.0 (GraphPad Software, San Diego, CA, USA) and JMP version 8 (SAS Institute, Cary, NC, USA) were used to analyze data. Results were expressed as mean ± SD. Statistical comparisons between two groups were performed using Student’s t test. The significance was adjusted to control for a false discovery rate at <5% for multiple apo parameter testing using the Benjamini–Hochberg procedure [44].

## Results

**Table 1** shows the characteristics of MetSyn patients and control subjects. By design, age and sex distributions were similar. Differences in BMI, triglyceride and HDL-cholesterol levels were a reflection of the different inclusion/exclusion criteria for these two groups of subjects. Although not selection criteria, the observed differences in blood pressure and glucose levels were expected co-morbidities characteristic of the MetSyn. Total and LDL cholesterol levels were elevated in patients versus the controls.

**Table 1:** Subjects characteristics (mean ± SD a).

Variable	Control (n = 14)	MetSyn (n = 56)
Age (years)	44.9±12.3	47.8±10.6
Male Sex (N, %)	9 (64%)	33 (60%)
BMI (kg/m^2^)	22.9±1.3	32.1±3.8***
TG (mg/dL)	76.4±22.5	215±99***
HDL-C (mg/dL)	55.9±8.6	42.2±8.2***
Total Chol (mg/dL)	174±32	202±45*
LDL-C (mg/dL)	108±26	131±37*
Systolic BP (mmHg)	112±9	132±12***
Diastolic BP (mmHg)	69±7	82±7***
Fasting Glucose (mg/dL)	85±8	100±12*
HGB A1c (%)	5.4±0.4	5.5±0.4
Smokers (N, %)	0 (0%)	4 (7%)
Anti-hypertensive Med (N, %)	0 (0%)	14 (25%)
Statins Use (N, %)	0 (0%)	8 (14%)

a unless noted otherwise; *p<0.05, **p<0.01, ***p<0.01 vs. control.

### 1D electrophoresis for the analysis of lipoprotein proteome

1D electrophoresis using 4–20% Tris-Tricine gradient gels allowed for simultaneous and consistent resolution of apos and their isoforms in a high throughput fashion. 210 lipoprotein preparations from a total of 70 participants were analyzed on 20 gels. Protein separation based on the molecular weight produced consistent band patterns in all gels **(Figure S1)**. 27 bands were measured in VLDL fractions, 29 in LDL, and 28 in HDL in all 70 subjects. Major bands were identified by comparing their migration patterns between 1D and 2D gels aided by LC-MS/MS identification and other methods **(Table S1 and Figure S2)**. The coefficient of variation was calculated for major HDL bands from one lab control sample, which was run on every gel **(Table S2)**. The coefficient of variation was lower for smaller proteins (apoA2, apoC2, and isoforms of apoC3; 4–6%) and higher for larger proteins (22–23% for apoE isoforms). The presence of differentially glycosylated apoC3 isoforms **(Figure S3A)** was confirmed by MALDI-TOF analysis of VLDL and HDL preparations, and in whole plasma from one subject (HDL results are shown in **Figure S3B**). In addition, sialylated isoforms of apoC3 were clearly sensitive to neuraminidase, confirming the presence of sialic acid residues in the higher MW apoC3 bands **(Figure S3C)**. A 140 kDa isoform of apoB, corresponding to the amino terminal portion of the molecule, was identified based on nanoLC-MS/MS “peptide coverage” (a mapping of all detected tryptic digest peptides onto full length apoB protein sequence) and by western blotting with an antibody specific to amino-terminal peptide epitope of human apoB **(Figure S4)**.

### Total plasma apolipoprotein concentrations

The total plasma concentrations of apos were determined from the results of quantitative 1D electrophoresis by summing the concentrations in VLDL, LDL, and HDL fractions of the sequential ultracentrifugation **(** **Table 2** **)**. Total plasma apoB, apoC2 and apoC3 were higher and apoA1 was lower in MetSyn patients compared to control subjects. There was also a reduction of SAA4 in plasma of MetSyn subjects compared to controls.

**Table 2:** Total plasma apolipoprotein levels (mg/dL plasma a) from scanned 1D gradient electrophoresis gels (mean ± SD).

Apolipoprotein	Control (n = 14)	MetSyn (n = 56)
apoA1	117.7±14.9	98.8±19.1**
apoA2	34.0±7.2	32.2±7.2
apoB	69.8±17.4	87.9±23.2**
apoC2	7.3±1.7	9.3±2.8*
apoC3	24.8±4.8	33.8±12.7*
apoE	7.9±2.7	8.2±2.6
SAA4	15.0±5.1	11.6±3.5**

a Calculated as the sum of all isoforms recovered in very low, low and high density lipoprotein fractions (VLDL, LDL and HDL, respectively). *p<0.05, **p<0.01 adjusted for multiple comparisons.

### Apolipoproteins in individual lipoprotein fractions

There were significantly higher levels of VLDL - apoB, apoC3, and apoE among MetSyn subjects, implying an increase in VLDL particle number **(** **Table 3** **)**. Other significant differences were the higher levels of LDL - apoC3 (sum of isoforms) and the lower levels of HDL- SAA4 (sum of isoforms) in the patients vs. the controls. The other significant findings were the decrease of HDL- apoA1 and LDL - apoA1, and an increase in LDL - apoC2 in plasma of MetSyn subjects compared to controls.

**Table 3:** Apolipoproteins (mg/dL plasma) in VLDL, LDL, and HDL (mean ± SD).

Apolipoprotein	Fraction	Control (n = 14)	MetSyn (n = 56)
apoA1	VLDL	0.2±0.1	0.5±0.4
apoA1	LDL	8.9±2.5	7.0±5.0
apoA1	HDL	108.6±12.8	91.2±17.6
apoA2	VLDL	n.d	n.d
apoA2	LDL	n.d	n.d
apoA2	HDL	34.0±7.2	32.2±7.2
apoB	VLDL	2.9±1.0	7.5±3.3*
apoB	LDL	65.7±17.2	78.1±22.2
apoB	HDL	1.1±1.1	2.3±2.2
apoC2	VLDL	1.0±0.3	2.8±1.2
apoC2	LDL	0.4±0.2	0.8±0.4
apoC2	HDL	6.0±1.4	5.8±2.0
apoC3^a^	VLDL	3.0±0.9	7.6±3.6*
apoC3^a^	LDL	2.8±1.9	6.7±3.9***
apoC3^a^	HDL	19.0±3.8	19.5±7.1
apoE^a^	VLDL	0.6±0.2	1.5±0.7*
apoE^a^	LDL	2.9±1.3	3.1±1.7
apoE^a^	HDL	4.4±2.3	3.6±1.1
SAA4	VLDL	0.1±0.1	0.6±0.3
SAA4	LDL	0.5±0.2	0.8±0.3
SAA4 a	HDL	14.3±5.2	10.3±3.4*

a calculated as sum of isoforms; *p<0.05; ***p<0.001 vs. control, significant after adjustment for multiple testing. Abbreviations as in Table 2.

### Analysis of apo composition in individual lipoprotein fractions

With respect to the per particle apo composition of each lipoprotein fraction (we used a surrogate for per-particle composition calculated as a ratio of individual apo/apoB in VLDL and LDL fractions, or as apo/apoA1 in HDL), no significant differences were found in VLDL fraction; LDL fractions from MetSyn subjects were enriched with apoC2 and with apoC3 (the total and mono-sialylated isoforms) and contained less apoA1 when compared to controls; HDL from MetSyn subjects were enriched with apoA2, with the mono-sialylated isoform of apoC3, and with the low MW SAA while showing a reduction in high MW apoE and high MW SAA4 isoforms in comparison to controls **(** **Table 4** **)**. Accordingly, the ratios of high/low MW isoforms of apoC3 (identified as di-sialo/mono-sialo), apoE and SAA4 were significantly lower in MetSyn subjects compared to controls across all lipoprotein classes **(** **Table 5** **)**.

**Table 4:** Apo composition of VLDL, LDL (µg/mg apoB), and HDL (µg/mg apoA1; mean ± SD).

Apolipoprotein	Control (n = 14)	MetSyn (n = 56)
VLDL	composition µg/mg apoB	
apoA1	75.6±75.2	72.8±50.0
apoB 140 kDa	4.8±3.7	10.0±10.4
apoC1	44.4±28.5	55.0±44.1
apoC2	378±224	424±306
apoC3 di-sialo	505±326	389±316
apoC3 mono-sialo	690±645	680±560
apoC3 non-glyco	109±136	114±147
(apoC3 sum of isoforms)	1304±1082	1184±1005
apoE high MW	78.8±35.1	63.6±49.9
apoE low MW	163±69.6	162±107
(apoE sum of isoforms)	242±103	226±154
SAA4 low MW	45.8±30.3	88.3±90.4
LDL	composition µg/mg apoB	
apoA1	124±49.5	80.4±52.2*
apoB 140 kDa	101±93.1	119±97.0
apoC1	6.4±3.6	5.7±2.3
apoC2	6.1±3.4	10.2±4.8**
apoC3 di-sialo	17.8±7.1	27.4±17.9
apoC3 mono-sialo	23.9±14.5	61.9±37.0**
(apoC3 sum of isoforms)	41.8±20.2	89.3±53.4**
apoE high MW	10.6±4.6	6.9±7.1
apoE low MW	32.5±9.3	35.4±25.3
(apoE sum of isoforms)	43.1±12.0	42.2±31.8
SAA4 low MW	8.1±3.4	10.0±3.6
HDL	composition µg/mg apoA1	
apoA2	311±49.8	354±46.9**
apoB	10.6±10.4	27.68±28.6
apoB 140 kDa	3.3±3.7	5.6±5.4
apoC1	9.8±5.3	10.0±4.3
apoC2	55.4±12.6	64.4±20.1
apoC3 di-sialo	69.0±14.3	64.3±27.9
apoC3 mono-sialo	106±22.8	150±48.0**
(apoC3 sum of isoforms)	175±27.8	214±71.0
apoE high MW	12.8±7.1	8.9±4.0*
apoE low MW	27.5±15.4	30.8±7.8
(apoE sum of isoforms)	40.3±21.9	39.7±10.4
SAA4 high MW	76.3±43.2	37.2±16.2**
SAA4 low MW	56.2±19.3	75.4±18.8**
(SAA4 sum of isoforms)	132±49.6	113±28.4

*p<0.05; **p<0.01 vs. control, significant after adjustment for multiple testing. MW = molecular weight; other abbreviations as in Table 2.

**Table 5:** ApoC3, apoE and SAA4 isoform ratios for in VLDL, LDL, and HDL density fractions (mean ± SD).

Isoform Ratio	Fraction	Control (n = 14)	MetSyn (n = 56)
apoC3: di-sialo/mono-sialo	VLDL	0.80±0.19	0.58±0.13**
	LDL	0.84±0.29	0.47±0.16***
	HDL	0.69±0.22	0.43±0.13**
apoE: high MW/low MW	VLDL	0.48±0.09	0.39±0.12**
	LDL	0.33±0.13	0.19±0.09**
	HDL	0.48±0.12	0.29±0.10***
SAA4: high MW/low MW	HDL	1.46±0.95	0.51±0.21***

Abbreviations as in Tables 2 and 4. *p<0.05; **p<0.01; ***p<0.001 vs. control, significant after adjustment for multiple testing.

## Discussion

The purpose of this study was to explore potential differences in the apo composition (amounts, species, and isoforms) of VLDL, LDL and HDL in MetSyn patients as compared with healthy controls. We utilized a relatively inexpensive and simple method - 1D gradient gel electrophoresis – as our primary research tool, and confirmed its results by comparison with more expensive and/or labor intensive methods (2D gels, MALDI-TOF, nanoLC-MS/MS, western blotting, and enzymatic assays).

The differences in whole plasma apo content in MetSyn patients and controls were not surprising considering differences in inclusion criteria between MetSyn and control groups (high TG and low HDL-C). As expected, we observed higher apoB and lower apoA1 (major apos of TG-rich and HDL particles, respectively). The apoB/apoA1 ratio in our study (0.59 in controls and 0.89 in MetSyn) essentially matches published assessments in a large cohort (0.69 and 0.90, respectively); this parameter was suggested to have a potential to discriminate MetSyn individuals with higher CVD risk [45], [46]. Plasma levels of apoC3 were also reported to be elevated in MetSyn similar to this study [16]. Apo differences generally tracked with differences in standard lipoprotein cholesterol levels, i.e., higher apoB is associated with higher total and LDL cholesterol, lower apoA1 associated with lower HDL cholesterol and higher apoC2 and apoC3 associated with higher VLDL. These data would suggest that it was only particle numbers that differed, not so much the apo composition of specific lipoprotein fractions between MetSyn patients and controls. Therefore, we tested the effect of MetSyn on apo/apoB ratios in VLDL and LDL and on apo/apoA ratios in HDL to address the apo composition **(** **Table 4** **)**.

There were 9 significant differences in the apo composition of LDL and HDL (the apo composition of VLDL did not differ between patients and controls). Specifically his study found that: apoA1 was depleted in LDL fraction from MetSyn subjects possibly reflecting a lower level of the very large, buoyant HDL subspecies associated with MetSyn [47]; LDL/IDL fractions from MetSyn subjects were enriched with apoC2 and apoC3, possibly due to the accumulation of VLDL remnant particles in MetSyn [48]; and HDL fractions from MetSyn subjects were enriched with apoA2, which may be associated with the increased number of LpAI/LpAII particles and altered HDL metabolism [49], [50].

The remaining five differences in LDL and HDL particle composition (out of 9 detected) were seen in isoforms of apoC3, apoE and SAA4 **(** **Table 4** **)**. The significant reductions in the ratios of high-to-low MW species of apoC3, apoE, and SAA4 were observed on all three lipoprotein classes **(** **Table 5** **)**. These differences in apo glycosylation were the novel findings of this study. ApoC3, apoE, and SAA4 are of smaller MW (relative to apoB, for example) and therefore their glycosylated isoforms were readily resolved. We cannot, however, exclude the possibility that the extent of glycosylation of larger MW apos might have been affected as well but were unresolved on the 1D gel. For example, variations in the extent of glycosylation (sialylation) of apoB could not be detected by our method. Although sialylation of apoB is known to contributes 8% to the total amount of sialic acid in VLDL (apoC3 contributes 68% of sialic acid of VLDL), in LDL, apoB carries 60% of the particle sialic acid pool [27]. For the same reason (higher MWs) we were not able to resolve individual sialo-isoforms of apoE and SAA4 in 1D electrophoresis.

About 50% of apoC3 is modified by O-linked glycosylation with a single sialic acid attached to the glycan chain (mono-sialylated isoform), 40% modified with O-linked glycan containing two sialic acid residues (di-sialylated isoform), while the remaining fraction is non-glycosylated [51]–[53]; about 80% of apoE has been reported to be non-glycosylated with the remainder modified by O-glycosylation containing sialic acid [23], [54]; and about 50% of constitutive SAA4 present in human non-acute phase HDL is modified by N-linked glycosylation giving rise to two isoforms of 14 and 19 kDa [29], [55], [56]. In our study we observed similar extents of glycosylation: (1) apoC3 was distributed 8%, 52%, and 40% between non-glycosylated, mono- and di-sialylated isoforms in VLDL from healthy subjects; whereas in MetSyn subjects 9% of VLDL apoC3 was non-glycosylated, 58% mono-sialylated, and 33% di-sialylated; (2) 76% and 68% of apoE was non-glycosylated in LDL and HDL in the healthy controls, whereas MetSyn subjects had a slightly increased percentage of non-glycosylated apoE –82% in LDL and 80% in HDL; (3) 57% of SAA4 was glycosylated in HDL from controls compared to only 33% from MetSyn subjects.

Of note, the study by Hiukka et al. found high levels of apoC3 sialylation in patients with diabetes compared with healthy controls [57]. About 85% of diabetic participants in this study were on oral antihyperglycemic treatment. The finding of increased apoC3 sialylation in this population was not surprising as it is consistent with a previous study in subjects with diabetes on metformin therapy by Harvey et al. [38]. Harvey et al. found that obese diabetics on metformin (who were eligible for bariatric surgery) displayed near normal ratio of apoC3 glycoisoforms. In contract apoC3 glycosylation in non-diabetic obese individuals (without metformin therapy) was reduced compared to healthy controls. Therefore Harvey et al. suggested that apoC3 sialylation is responsive to metformin therapy. In our own experience (unpublished data) apoC3 sialylation is also responsive to the combination therapy with prescription omega-3 fatty acids and niacin. Therefore it is possible that sialylation of apoC3 in Hiukka et al. study of diabetic patients was affected by their medications. Further studies are needed to address apoC3 glycosylation state in diabetic patients in the absence of metformin therapy.

Interestingly, recent genome-wide association studies (GWAS) found that the GALNT2 gene, which belongs to a family of genes encoding enzymes catalyzing the first step of O-glycosylation, was associated with elevated serum TG and reduced HDL-C levels [58], [59]. Among known targets of GALNT2-catalyzed glycosylation are proteins that regulate lipoprotein metabolism: angiopoietin-like 3 (Angptl3), apoC3, and apoE [25], [60]. Although GALNT2 activity is redundant (its family contains 20 enzymes with overlapping activity), it is plausible that such an enzyme could play a role in the regulation of apo glycosylation in MetSyn. On the other hand, it is possible that apo carbohydrate content is (also) modifiable while in circulation. To this end, sialydases capable of trimming terminal sialic acid residues, NEU1 and NEU3, were identified on erythrocyte membranes [61]. The significance of apo glycosylation in disease remains to be established. Some data suggest that de-sialylation of human LDL by plasma trans-sialidase activity makes these modified LDL particles more atherogenic since de-sialylated LDL led to a greater cholesteryl ester accumulation in human aortic intimal smooth muscle cells [62]. Mechanistically, higher sialic acid levels in LDL might block the interaction of the particle with arterial wall proteoglycans, linking LDL hypo-glycosylation with atherogenesis [34], [63].

Two strengths of the study were the careful selection of both the (optimally) healthy controls and the MetSyn subjects [64], [65] and the use of an inexpensive (but highly reproducible) method, 1D electrophoresis. This method allowed for simultaneous quantitation of apos in a high throughput fashion. However, low sensitivity on the method (2 ng protein) compared to the high sensitivity of quantitative LS/MS methods [66] precluded us from the analysis of less abundant proteins and their isoforms associated with lipoproteins. Moreover, 1D gradient electrophoresis has a limited capacity to resolve protein isoforms compared to 2D electrophoresis and mass-spectrometry methods. Lastly, another technical limitation of our study was the use of high ionic strength solutions in the density ultracentrifugation method. It has been reported that high ionic strength used for isolation of lipoproteins with potassium bromide (KBr, used in our study) and sodium iodide could alter the retention of associated exchangeable proteins [42] therefore some important differences in LDL and HDL associated apolipoproteins could have been neglected.

In conclusion, we have observed a diminished glycosylation of three lipoprotein-associated apos – apoC3, apoE and SAA4– in patients with MetSyn. The clinical implications of this alteration in apo biochemistry remain to be elucidated.

## Supporting Information

Figure S1 **1D gel electrophoresis.** 4.5 µg of VLDL, LDL, and HDL were resolved on 4–20% Tris-Tricine peptide gel. Representative preparations (from one individual).
(TIF)

Figure S2 **Comparison of 1D and 2D gel electrophoresis patterns.** VLDL (**A**) and HDL (**B**) samples were resolved by 2D gel electrophoresis and compared to (i) the results of 1D gel electrophoresis of the same samples (right sub-panels) and to (ii) previously identified proteins found in published images of 2D electrophoretic separation of VLDL and HDL. Arrows and labels in 2D gels indicate apo bands, which are consistent with published data [1], [2]. Arrows in 1D subpanels indicate apo bands, which were directly inferred from their migration in 2D gels and confirmed by mass-spectrometry (Table S1). 1. Sun HY, Chen SF, Lai MD, Chang TT, Chen TL, et al. (2010) Comparative proteomic profiling of plasma very-low-density and low-density lipoproteins. Clin Chim Acta 411∶336–344. 2. Stahlman M, Davidsson P, Kanmert I, Rosengren B, Boren J, et al. (2008) Proteomics and lipids of lipoproteins isolated at low salt concentrations in D2O/sucrose or in KBr. J Lipid Res 49∶481–490.
(TIF)

Figure S3 **Identification of ApoC3 glycoisoforms. A.** Differentially glycosylated ApoC3 isoforms; GalNAc, N-Acetylgalactosamine; Gal, galactose; NeuAc, N-Acetylneuraminic Acid or Sialic Acid; **B.** HDL analyzed by MALDI-TOF; arrows and labels point to apoC3 and apoC2 peaks, which relative intensities and masses are consistent with published data [1]; **C.** VLDL, and **D.** HDL preparation were treated with neuraminidase to remove terminal sialic acid residues and analyzed by 1D electrophoresis followed by Coomassie staining. 1. Harvey SB, Zhang Y, Wilson-Grady J, Monkkonen T, Nelsestuen GL, et al. (2009) O-glycoside biomarker of apolipoprotein C3: responsiveness to obesity, bariatric surgery, and therapy with metformin, to chronic or severe liver disease and to mortality in severe sepsis and graft vs. host disease. J Proteome Res 8∶603–612.
(TIF)

Figure S4 **Identification of 140 kDa apoB isoform by LC-MS/MS and western blotting. A.** Tryptic digest peptides (vertical lines) detected in LDL apoB band and HDL 140 kDa band were mapped on the human apoB protein sequence (aa 1–4563); **B.** western blotting of LDL and HDL fractions with antibody specific to amino-terminal epitope from human apoB.
(TIF)

Table S1 Major apolipoproteins identified by 1D gradient gel electrophoresis in three lipoprotein fractions and confirmation methodology.
(DOCX)

Table S2 Technical variation of HDL band intensity measurements.
(DOCX)
