# Supplementary figures and images for: Reduced Apolipoprotein Glycosylation in Patients with the Metabolic Syndrome
Source: PLoS One. 2014 Aug 12;9(8):e104833. doi: 10.1371/journal.pone.0104833 (PMC4130598; doi:10.1371/journal.pone.0104833)

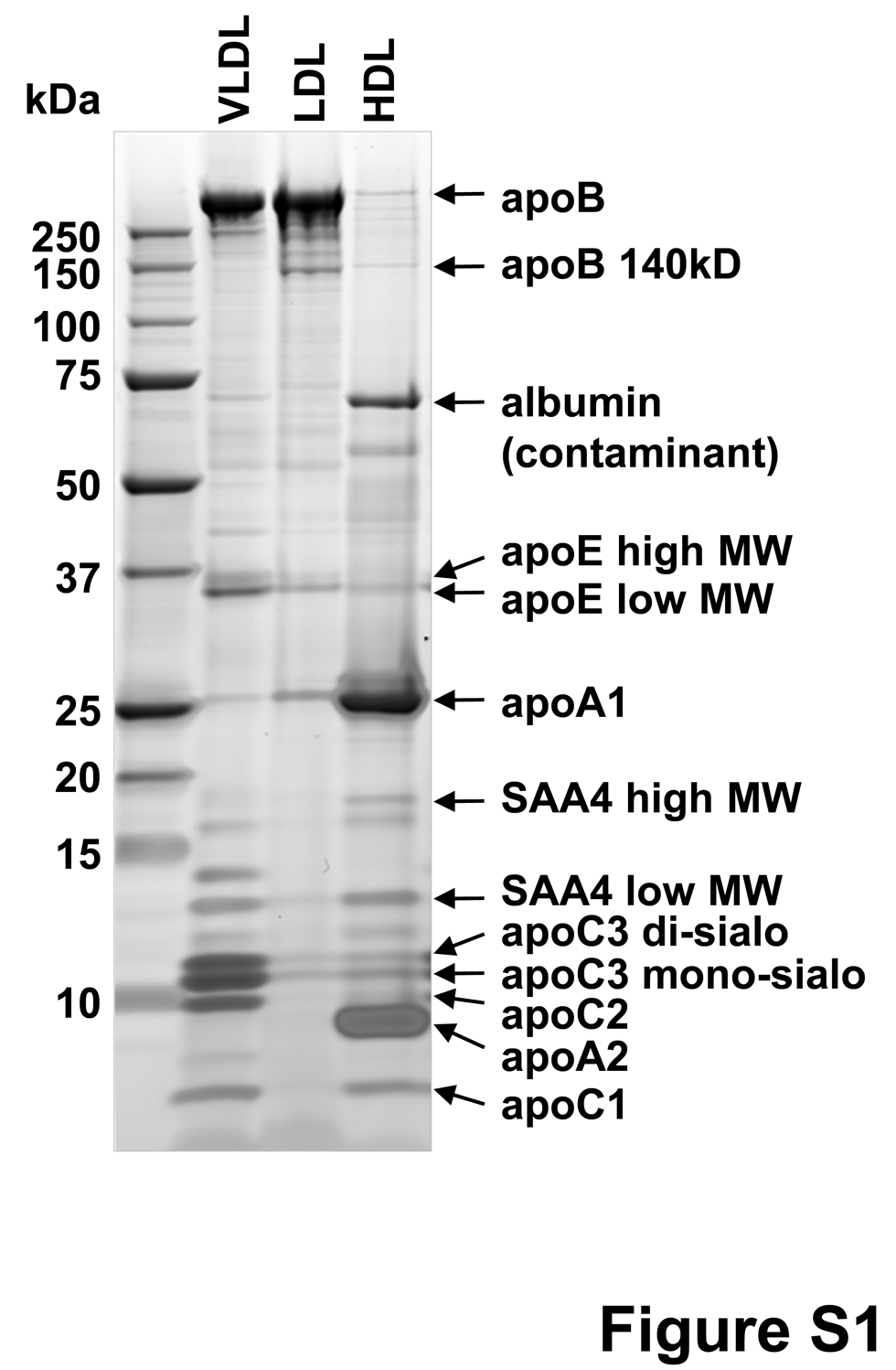

Supplement: Figure S1 — 1D gel electrophoresis. 4.5 µg of VLDL, LDL, and HDL were resolved on 4–20% Tris-Tricine peptide gel. Representative preparations (from one individual). (TIF) [file pone.0104833.s001.tif]

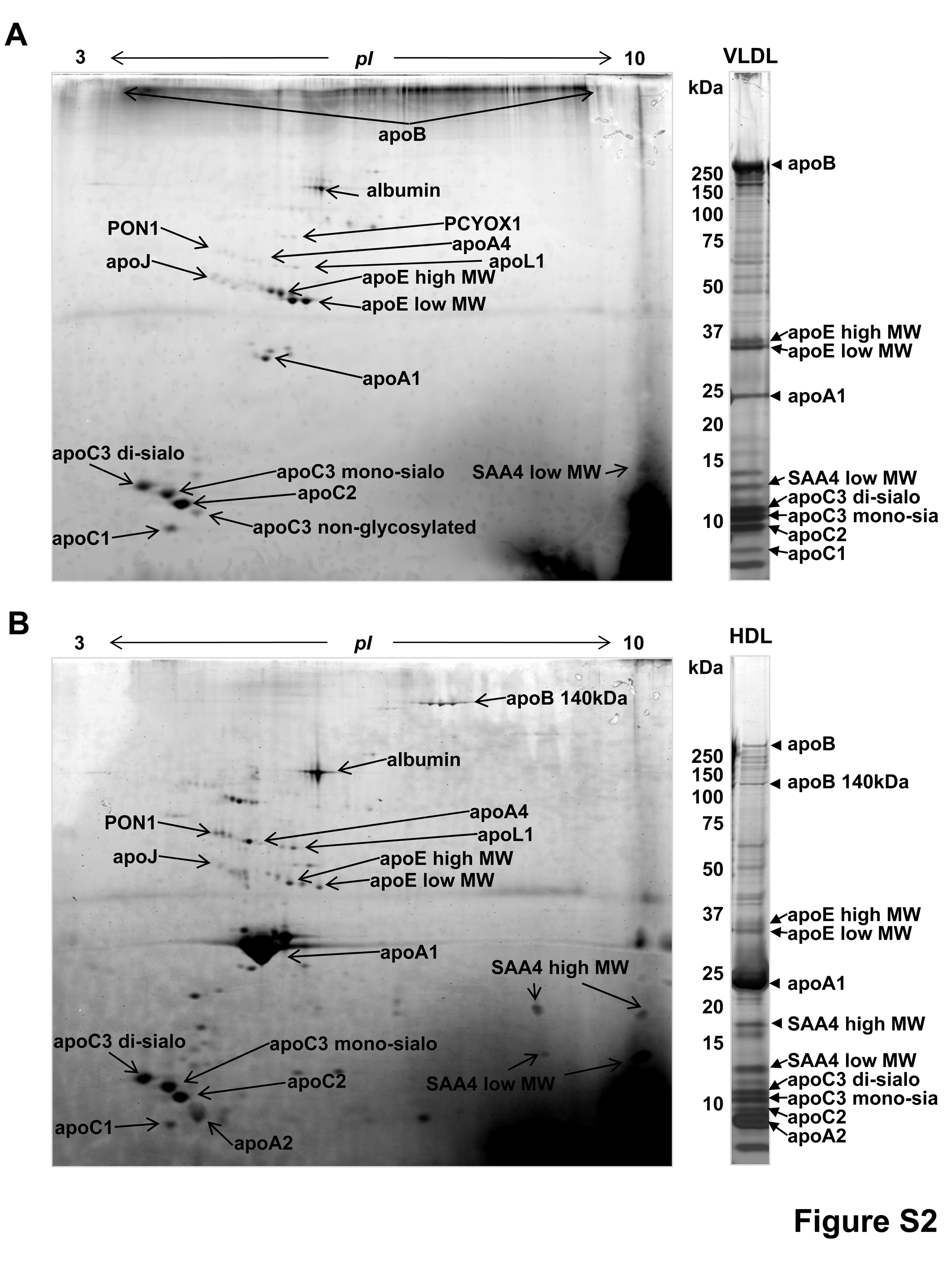

Supplement: Figure S2 — Comparison of 1D and 2D gel electrophoresis patterns. VLDL (A) and HDL (B) samples were resolved by 2D gel electrophoresis and compared to (i) the results of 1D gel electrophoresis of the same samples (right sub-panels) and to (ii) previously identified proteins found in published images of 2D electrophoretic separation of VLDL and HDL. Arrows and labels in 2D gels indicate apo bands, which are consistent with published data [1], [2]. Arrows in 1D subpanels indicate apo bands, which were directly inferred from their migration in 2D gels and confirmed by mass-spectrometry (Table S1). 1. Sun HY, Chen SF, Lai MD, Chang TT, Chen TL, et al. (2010) Comparative proteomic profiling of plasma very-low-density and low-density lipoproteins. Clin Chim Acta 411∶336–344. 2. Stahlman M, Davidsson P, Kanmert I, Rosengren B, Boren J, et al. (2008) Proteomics and lipids of lipoproteins isolated at low salt concentrations in D2O/sucrose or in KBr. J Lipid Res 49∶481–490. (TIF) [file pone.0104833.s002.tif]

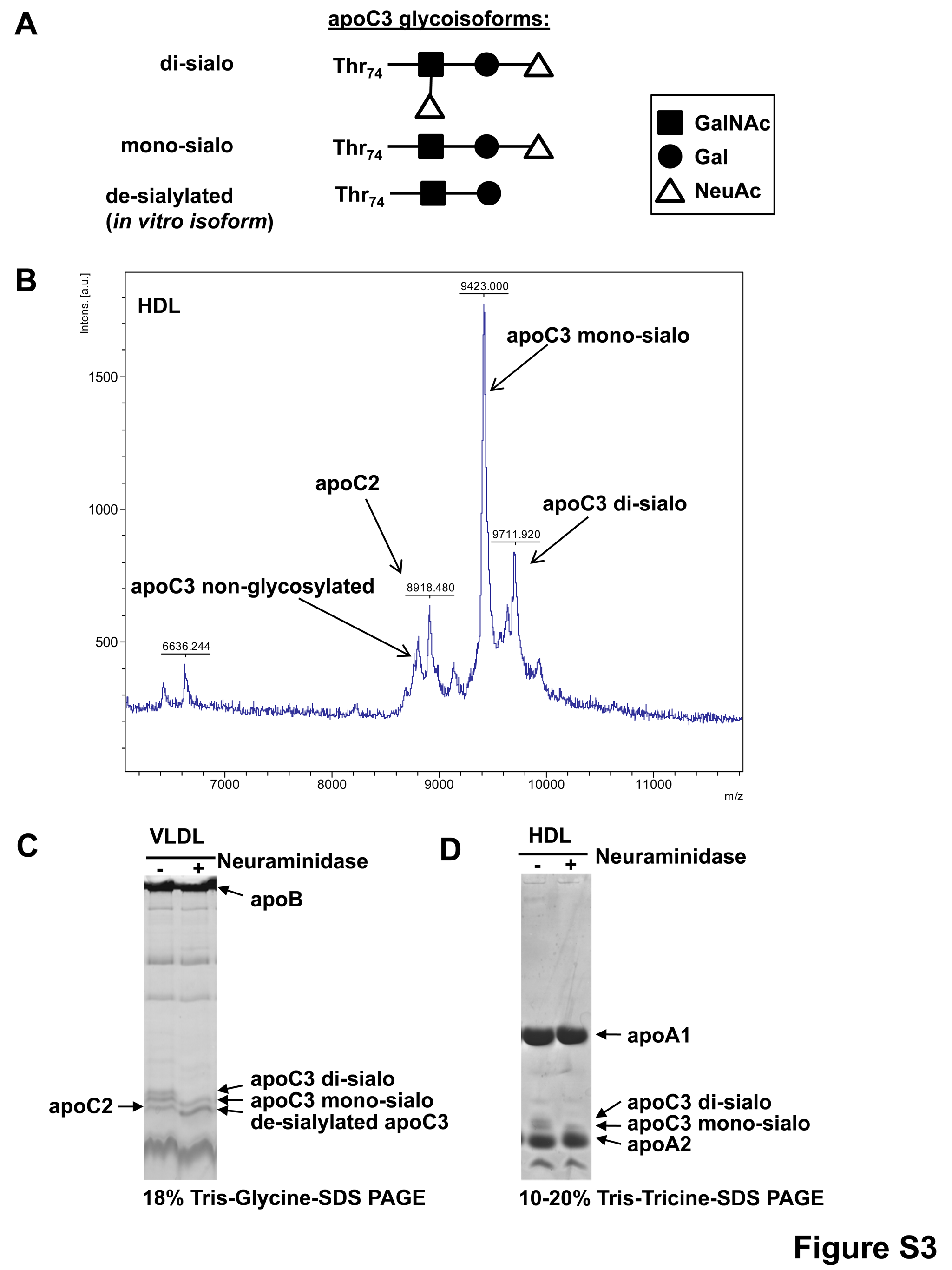

Supplement: Figure S3 — Identification of ApoC3 glycoisoforms. A. Differentially glycosylated ApoC3 isoforms; GalNAc, N-Acetylgalactosamine; Gal, galactose; NeuAc, N-Acetylneuraminic Acid or Sialic Acid; B. HDL analyzed by MALDI-TOF; arrows and labels point to apoC3 and apoC2 peaks, which relative intensities and masses are consistent with published data [1]; C. VLDL, and D. HDL preparation were treated with neuraminidase to remove terminal sialic acid residues and analyzed by 1D electrophoresis followed by Coomassie staining. 1. Harvey SB, Zhang Y, Wilson-Grady J, Monkkonen T, Nelsestuen GL, et al. (2009) O-glycoside biomarker of apolipoprotein C3: responsiveness to obesity, bariatric surgery, and therapy with metformin, to chronic or severe liver disease and to mortality in severe sepsis and graft vs. host disease. J Proteome Res 8∶603–612. (TIF) [file pone.0104833.s003.tif]

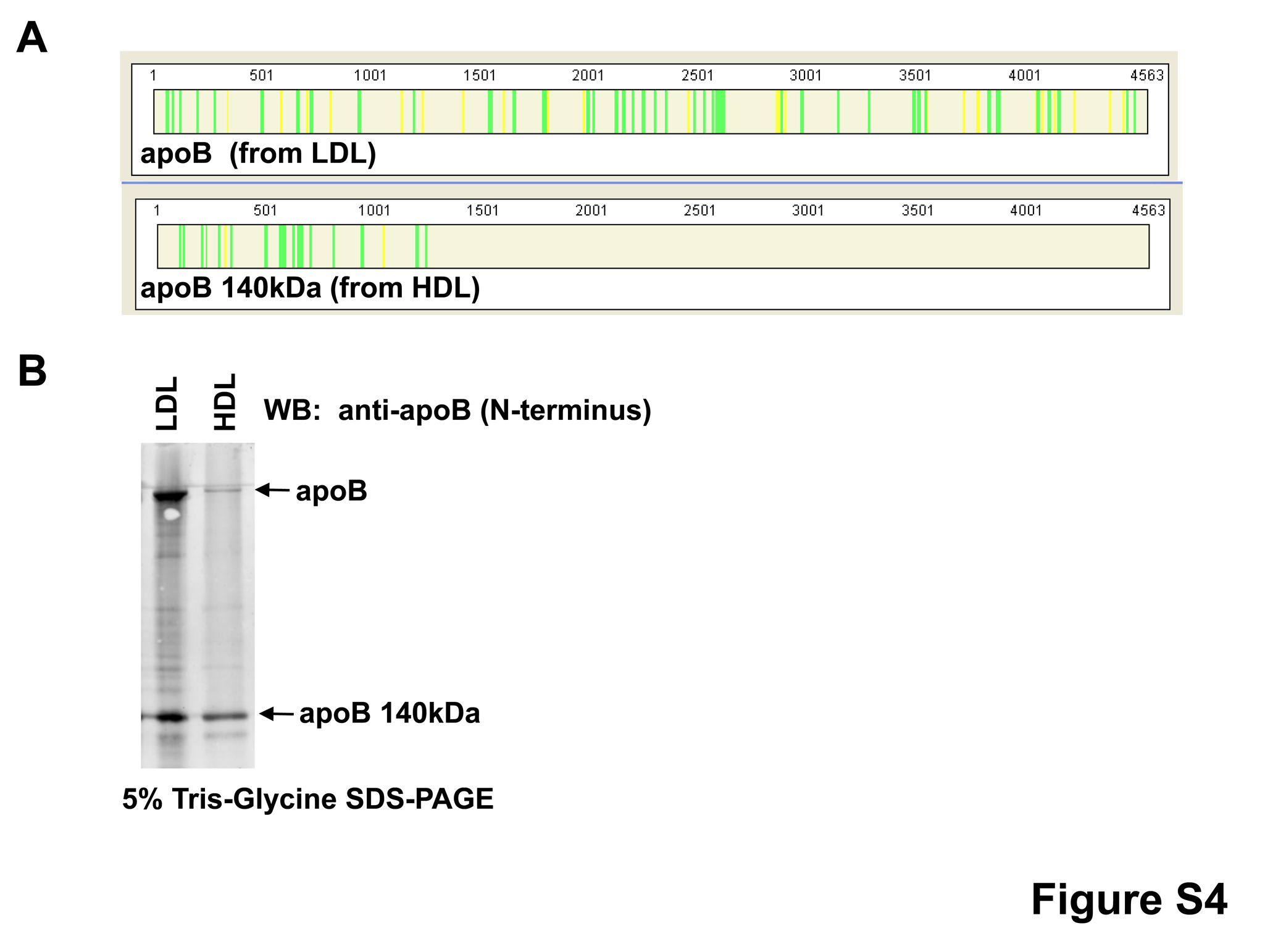

Supplement: Figure S4 — Identification of 140 kDa apoB isoform by LC-MS/MS and western blotting. A. Tryptic digest peptides (vertical lines) detected in LDL apoB band and HDL 140 kDa band were mapped on the human apoB protein sequence (aa 1–4563); B. western blotting of LDL and HDL fractions with antibody specific to amino-terminal epitope from human apoB. (TIF) [file pone.0104833.s004.tif]
