# Supplementary material for: Reduced Apolipoprotein Glycosylation in Patients with the Metabolic Syndrome
Source: PLoS One. 2014 Aug 12;9(8):e104833. doi: 10.1371/journal.pone.0104833 (PMC4130598; doi:10.1371/journal.pone.0104833)
Supplement: Table S1 — Major apolipoproteins identified by 1D gradient gel electrophoresis in three lipoprotein fractions and confirmation methodology. (DOCX) [file pone.0104833.s005.docx]

**Table S1.** Major apolipoproteins identified by 1D gradient gel electrophoresis in three lipoprotein fractions and confirmation methodology

| Apolipoprotein | VLDL | LDL | HDL | ID Confirmation |
| --- | --- | --- | --- | --- |
| apoA1 | + | + | + | LC-MS/MS ^a,c^ |
| apoA2 |  |  | + | LC-MS/MS ^c^ |
| apoB | + | + | + | LC-MS/MS ^a^; WB ^b.c^ |
| apoB 140kDa | + | + | + | LC-MS/MS ^c^; WB ^b,c^ |
| apoC2 | + | + | + | LC-MS/MS ^a,c^ |
| apoC3 di-sialo | + | + | + | LC-MS/MS ^a,c^; MALDI-TOF ^a,c^ |
| apoC3 mono-sialo | + | + | + | LC-MS/MS ^a,c^; MALDI-TOF ^a,c^ |
| apoC3 non-glycosylated | + |  | n.r. | MALDI-TOF ^a,c^ |
| apoE high MW | + | + | + | LC-MS/MS ^c^ |
| apoE low MW | + | + | + | LC-MS/MS ^a^ |
| SAA4 high MW |  |  | + | LC-MS/MS ^c^ |
| SAA4 low MW | + | + | + | LC-MS/MS ^c^ |

+, detected in all subjects; n.r., not resolved (masked by apoA2 band); ID conformation was obtained in (^a^) VLDL, (^b^) LDL, or (^c^) HDL fractions; MW, molecular weight; LC-MS/MS, liquid chromatography – tandem mass spectrometry; MALDI-TOF, Matrix Assisted Laser Desorption Ionization Time-of-Flight mass spectrometry.
