# Supplementary material for: Reduced Apolipoprotein Glycosylation in Patients with the Metabolic Syndrome
Source: PLoS One. 2014 Aug 12;9(8):e104833. doi: 10.1371/journal.pone.0104833 (PMC4130598; doi:10.1371/journal.pone.0104833)
Supplement: Table S2 — Technical variation of HDL band intensity measurements. (DOCX) [file pone.0104833.s006.docx]

**Table S2.** Technical variation of HDL band intensity measurements

| HDL band (n=20 replicas ^a^) | Raw band intensity ^b^, a.u. | Coefficient of variation ^c^, % |
| --- | --- | --- |
| apoA1 | 46206 ± 1174 | 11.4 |
| apoA2 | 26080 ± 3919 | 5.8 |
| apoC1 | 18330 ± 4322 | 15.5 |
| apoC2 | 21848 ± 3951 | 5.0 |
| apoC3 di-sialo | 23119 ± 4042 | 4.9 |
| apoC3 mono-sialo | 28340 ± 4553 | 4.2 |
| apoE high MW | 9262 ± 2683 | 21.9 |
| apoE low MW | 7687 ± 2180 | 22.7 |
| SAA4 high MW | 6088 ± 1541 | 14.8 |
| SAA4 low MW | 10943 ± 2591 | 10.8 |

^a^ from 20 SDS-PAGE gels; ^b^ mean ± SD; ^c^ coefficient of variation was calculated based on raw band intensities normalized to total lane intensity; a.u. arbitrary units of fluorescent intensity
